# Understanding the impact of the cofactor swapping of isocitrate dehydrogenase over the growth phenotype of *Escherichia coli* on acetate by using constraint-based modeling

**DOI:** 10.1371/journal.pone.0196182

**Published:** 2018-04-20

**Authors:** Erick Armingol, Eduardo Tobar, Ricardo Cabrera

**Affiliations:** Laboratorio de Bioquímica y Biología Molecular, Facultad de Ciencias, Universidad de Chile, Santiago, Chile; Universite Paris-Sud, FRANCE

## Abstract

It has been proposed that NADP^+^-specificity of isocitrate dehydrogenase (ICDH) evolved as an adaptation of microorganisms to grow on acetate as the sole source of carbon and energy. In *Escherichia coli*, changing the cofactor specificity of ICDH from NADP^+^ to NAD^+^ (cofactor swapping) decreases the growth rate on acetate. However, the metabolic basis of this phenotype has not been analyzed. In this work, we used constraint-based modeling to investigate the effect of the cofactor swapping of ICDH in terms of energy production, response of alternative sources of NADPH, and partitioning of fluxes between ICDH and isocitrate lyase (ICL) -a crucial bifurcation when the bacterium grows on acetate-. We generated *E*. *coli* strains expressing NAD^+^-specific ICDH instead of the native enzyme, and bearing the deletion of the NADPH-producing transhydrogenase PntAB. We measured their growth rate and acetate uptake rate, modeled the distribution of metabolic fluxes by Flux Balance Analysis (FBA), and quantified the specific activities of NADPH-producing dehydrogenases in central pathways. The cofactor swapping of ICDH led to one-third decrease in biomass yield, irrespective of the presence of PntAB. According to our simulations, the diminution in growth rate observed upon cofactor swapping could be explained by one-half decrease in the total production of NADPH and a lower availability of carbon for biosynthesis because of a change in the partition at the isocitrate bifurcation. Together with an increased total ATP production, this scenario resulted in a 10-fold increment in the flux of ATP not used for growing purposes. PntAB was identified as the primary NADPH balancing response, with the dehydrogenases of the oxidative branch of the Pentose Phosphate Pathway and the malic enzyme playing a role in its absence. We propose that in the context of *E*. *coli* growing on acetate, the NADP^+^-specificity of ICDH is a trait that impacts not only NADPH production, but also the efficient allocation of carbon and energy.

## Introduction

Heterotrophic bacteria oxidize available carbon sources generating NADPH and NADH through the dehydrogenases of the central metabolic pathways. Dehydrogenases usually present a strong segregation in their cofactor specificity [1]. In *Escherichia coli* (*E*. *coli*), the isocitrate dehydrogenase (ICDH) from the TriCarboxylic Acids (TCA) cycle is an NADP^+^-specific enzyme that provides most of NADPH when the bacterium uses acetate as the sole source of carbon and energy [2]. Conversely, ICDH is NAD^+^-specific in microorganisms that cannot grow on acetate [3].

A balance between the production and consumption of NADH and NADPH is needed to keep the cellular homeostasis. Not surprisingly, different mechanisms have been described to enable this balance, such as the presence of transhydrogenases [4]. *E*. *coli* expresses two of these enzymes: the membrane-bound PntAB and the cytosolic UdhA. They rebalance the insufficiency or excess of catabolic production of NADPH, by producing (PntAB) or consuming (UdhA) this cofactor, respectively [4, 5]. When *E*. *coli* grows on acetate, the high carbon flux through ICDH leads to an overproduction of NADPH [2], which is fundamentally countered by UdhA [5].

It has been proposed that the specificity of ICDH for NADP^+^ has evolved as an adaptation to grow on acetate [6]. By using an engineered NAD^+^-dependent ICDH, Zhu *et al*. [3] demonstrated that the production of NADH -instead of NADPH- by this metabolic reaction negatively impacts the growth rate of *E*. *coli* on acetate. The negative effect was more pronounced in the absence of PntAB and the malic enzyme (alternative sources of NADPH). Conversely, the cofactor swapping of ICDH increased the growth rate in the absence of UdhA. Unfortunately, growth rate does not provide by itself a picture of the effect of the metabolic perturbation on the energetic efficiency of converting an energy-poor substrate such as acetate into biomass. In regard to specific responses of rebalancing NADPH deficiency, Wang *et al*. [7] observed that the activity of NADP^+^-specific malic enzyme in extracts was almost 3.9-fold enhanced upon ICDH cofactor swapping. They highlighted the importance of this enzyme in providing NADPH for the growth of *E*. *coli* on acetate. In the case of PntAB, its contribution level has not been clarified with respect to the metabolic response of *E*. *coli* after cofactor swapping of ICDH.

When *E*. *coli* grows on acetate, a fraction of the isocitrate is diverted towards isocitrate lyase (ICL), bypassing ICDH. As this shunt avoids the oxidative decarboxylation reactions of the TCA cycle, it replenishes intermediates that are used for the biosynthesis of cellular constituents [8]. In fact, *E*. *coli* mutants lacking ICL activity lose the capacity to grow on acetate [9]. Interestingly, other organisms whose genome does not express ICL bear an NAD^+^-specific ICDH and they are unable to grow on acetate [3]. Then, for *E*. *coli* growing on acetate, the flux ratio at the isocitrate bifurcation must be finely regulated to obtain both a proper separation of the carbon available for biosynthesis from that lost as CO_2_ and a suitable production of NADPH. This regulation is achieved through phosphorylation of ICDH, which renders the enzyme inactive, decreasing the flow of isocitrate through the TCA cycle and so forcing it through ICL instead [10, 11]. LaPorte *et al*. [12] analyzed how the partitioning coefficient in a bifurcation responds to the rate of production of the common substrate and the kinetic parameters of the competing enzymes. They observed that modest changes in the maximum velocity of ICDH and the rate of isocitrate production dramatically altered the flux through ICL, as seen when the bacterium is changed from acetate to glucose. El-Mansi *et al*. [13] analyzed a wider network including the reactions of the TCA cycle and glyoxylate shunt, and constrained the calculations by using previously reported values of metabolite production for biomass synthesis. They observed that the decrement of ICDH concentration should increase the flux control coefficient of the reaction that this enzyme catalyzes. Expanding the analysis to the rest of the central pathways or the comprehensive metabolic network would provide a framework to understand the impact of flux partitioning at the isocitrate bifurcation on the growth rate and the biomass yield, but this has not been attempted. In addition, how sensitive is the flux partitioning towards the cofactor specificity of ICDH has not been addressed before.

One crucial aspect to understand the effect of changing the cofactor specificity of ICDH in *E*. *coli* is the analysis of its metabolic fluxes. In fact, characterizing the metabolic flux response after replacing the native dehydrogenase has been the focus of other studies of cofactor swapping [1, 14–16]. In this regard, Flux Balance Analysis (FBA) uses physiological parameters as constraints to model a feasible flux distribution that if occurs, it results in the observed phenotype [17, 18]. Hence, FBA may provide a systemic understanding of how ICDH cofactor swapping affects the phenotype of *E*. *coli*, including metabolic changes such as the production of energy, the response of alternative sources of NADPH, and the partitioning of fluxes at the isocitrate bifurcation. In the present study, we used this approach to analyze the metabolic impact of changing the cofactor specificity of ICDH in the presence and absence of PntAB. We first characterized the exponential phase of aerobic growth on acetate of wild type and mutant strains bearing these modifications. Using the rates of growth and acetate uptake as constraints, we modeled optimal and sub-optimal distributions of metabolic fluxes by FBA and Markov chain Monte Carlo (MCMC) sampling, respectively. Finally, we measured the specific activity of key dehydrogenases in cell extracts of the cultured strains to evaluate possible responses to ICDH cofactor swapping at this level.

## Material and methods

### Generation of mutant strains

Each genetic modification was performed using a previously described homologous recombination method, which employs a PCR fragment to replace a native gene in the chromosome [19]. The linear DNA molecule used to change the *icd* gene has the FRT-kan^R^-FRT cassette just downstream of the previously described encoding sequence of an NAD^+^-dependent *E*. *coli* ICDH (*icd*^*NAD*^ gene) [3]. This fragment (icd^NAD^-FRT-kan^R^-FRT) was amplified from the plasmid pUC57-icd^NAD^-FRT-kan^R^-FRT with *ad hoc* primers containing nucleotide sequences matching the chromosome 30 bp upstream and 30 bp downstream of the 1251 bp native sequence of ICDH. Likewise, the deletion of the *pntAB* operon was performed by amplifying the FRT-kan^R^-FRT cassette from the plasmid pKD13 with *ad hoc* primers for the homologous recombination.

Each fragment was purified and introduced into freshly prepared electrocompetent cells from the parental strain MG1655, transformed previously with plasmid pKD46 [19]. To accomplish recombination, cells were grown to OD_600_ 0.4–0.5 at 30°C and the expression of the Red recombinase system was induced with the addition of 10 mM arabinose for 1 h before transformation. The colonies obtained from the transformation using each fragment correspond to *icd*^*NAD*^*-kan*^*R*^ and *ΔpntAB-kan*^*R*^ phenotypes, respectively. Chromosomal fragments inserted in the kanamycin-resistant colonies were moved into fresh wild type isolates of *E*. *coli* using a previously described backcross method [20, 21]. The double mutant strain (*icd*^*NAD*^ *ΔpntAB*) was obtained by using the *icd*^*NAD*^ strain as the fresh isolate of *E*. *coli* for the backcross procedure and the *ΔpntAB-kan*^*R*^ strain as donor. To detect the strains of interest, colony PCR for both *icd* and *pntAB* regions was used. In addition, sequencing of these regions was performed to confirm the modifications.

### Growth conditions and sampling

During genetic manipulations the strains were grown in liquid or solid LB rich medium, supplemented with *ad hoc* antibiotics. Wild type *E*. *coli* MG1655 and mutants derived thereof were used in all physiological experiments. All physiological experiments were performed by growing the cells in full aerobic conditions, in 500 mL flasks containing 100 mL M9 minimal medium supplemented with 3.0 g × L^-1^ of sodium acetate as the sole carbon source at 37°C on a gyratory shaker at 200 rpm. M9 minimal medium was prepared according to a previous report [22]. All experiments were performed in biological triplicate.

To determine the growth rate, samples were taken every 60 min to measure the OD_600_. To determine the biomass yield and calculate the acetate uptake rate, 6 samples were taken throughout the exponential phase, filtered using a 0.22 μm filter and immediately chilled at -80°C until analysis.

### Analytical procedures

The concentration of acetate in the filtrates was determined by HPLC (Waters 1525) using an Aminex HPX-87H ion exchange carbohydrate-organic acid column (Bio-Rad) at 65°C and a UV detector (Waters 2487, λ = 210 nm). The degassed mobile phase contained 5 mM H_2_SO_4_ and was run at a flow rate of 0.6 mL x min^-1^. The OD_600_ to dry weight conversion coefficient (ƒ) was determined gravimetrically after centrifuging at 3,215 x g samples of 15 mL obtained from cultures of wild type *E*. *coli* (taken throughout the culture), washing with distilled water, and drying at 100°C to a constant weight. Thus, the value of ƒ used in this work was 0.40 ± 0.01 gDW × L^-1^ × OD^-1^.

### Flux balance analysis

The comprehensive genome-scale model of *E*. *coli* iJO1366 was used as the wild type default Systems Biology Markup Language model [23]. Modeling was performed by solving FBA problems with targeted constraints. Generally, the FBA problem is a linear optimization problem, in which the solution that maximizes or minimizes an objective function (**Z**) is identified. This linear programming (LP) problem is formulated as:

$$Min\;Z=c^{T}∙v$$ (1)

Subject to:

S∙v=0 (2)

$$v_{i-lb}<v_{i}<v_{i-ub}$$ (3)

where **c** is the objective vector containing the reaction to be maximized or minimized, **v** is the vector of fluxes, **S** is the stoichiometric matrix, and **v**_**i-lb**_ and **v**_**i-ub**_ are the lower and upper bounds of the velocity of the **i-th reaction**, respectively. The formalism of FBA was previously described in detail [24].

Additional constraints to model the effects of genetic modifications were added. The lower and upper bounds of reactions catalyzed by deleted genes were changed to zero. To model the effects of changing the cofactor specificity of ICDH, the stoichiometric coefficients of NADP^+^ and NADPH of the reaction ICDHyr were interchanged with those of NAD^+^ and NADH, respectively. Additionally, the pyruvate:ferredoxin oxidoreductase (POR5) was made irreversible by setting to zero its lower bound, similarly to a previous method [25]. All the computational simulations were performed with COBRA Toolbox 2.0.5 [26] in MATLAB (MathWorks, Inc.), using GUROBI 5 (Gurobi Optimization, Inc.) as optimizer software. Flux solutions were calculated by using an iterative optimization method. Also, by performing Flux Variability Analysis (FVA) we identified the range of variation of every reaction, i.e. their minimal and maximal fluxes.

### Sensitivity analyses

On one hand, a robustness analysis was performed to assess the potential of the strains to produce ATP at different growth rates. On the other hand, a phenotype phase plane analysis was done to explore the behavior of biomass yield (z-axis) as a function of the production of isocitrate (x-axis) and the partition of flux between ICL and ICDH (y-axis). This analysis was taken on the wild type strain and the mutant bearing the NAD^+^-dependent ICDH, after constraining the ATPM flux (reaction that drains ATP from the model) to its optimal value, representing the maximal ATP yield for the modeling condition. There on, a robustness analysis was performed after constraining the production of isocitrate to its optimal value. In this case, we observed the response of biomass yield (y-axis) as a function of the partition of flux towards ICL (x-axis). All these analyses were implemented as described in [24].

### Iterative optimization

A procedure was designed to calculate a unique optimal flux distribution after constraining the models with the rates of growth and acetate uptake, both measured experimentally. The obtained distribution is intended to comply the following criteria: *i*. maximization of the ATP yield along with minimization of the sum of fluxes (according to Schuetz *et al*. [27] the metabolic optimality is defined by the sum of fluxes, the ATP yield and the biomass yield); *ii*. minimization of the oxygen uptake rate (which was not measured experimentally); and *iii*. elimination of loops (in order to perform further calculations such as the total production/consumption of metabolites, this criterion reduces the number of fluxes biologically unfeasible after setting a threshold). This method is essentially iterative, and was performed as follows:

1. The constraints of ATPM were removed (setting the lower and upper bounds to 0 and 50 mmol × gDW^-1^ × h^-1^, respectively).
2. The reaction ATPM was set up as the objective function to maximize the ATP yield by solving the classical FBA LP problem [24].
3. The resulting value was used to constrain the lower bound of ATPM.
4. The oxygen uptake rate was minimized by solving the classical FBA LP problem.
5. The resulting value was used to constrain the lower and upper bounds of EX_o2(e).
6. The reaction ATPM was set up as the objective function again.
7. The enumeration of the first 400 optima of maximal ATP yield was performed using MILP [28].
8. A flux distribution from the 400 optima was selected by identifying the one which minimizes the sum of squares of velocities (**v**_**i**_) from a total of **n** reactions: $\sum_{i=1}^{n}{v_{i}}^{2}$
9. For those reactions whose velocity was below a threshold (in this case, 500 mmol × gDW^-1^ × h^-1^), the lower and upper bounds were both set to the fluxes obtained in the previous step. Thus, after performing the remaining steps, it should be obtained a flux distribution with lower values of flux for those reactions that exceeded the threshold.
10. The maximization of the flux of ATPM was performed by using MILP. Again, 400 optima were calculated.
11. Finally, a flux distribution from the 400 optima was selected by identifying the one which minimizes the sum of squares of velocities.

### Net flux of consumption or production of metabolites

At steady state, the net flux of consumption or production (*φ*) of the **i-th** metabolite was calculated using the following formula:

$$\varphi_{i}=\frac{1}{2}\sum_{j=1}^{n}\left|S_{\left(i,j\right)}∙v_{j}\right|$$ (4)

Where **S(i, j)** is the stoichiometric coefficient of the **i-th** metabolite in the **j-th** reaction, and **v**_**j**_ is the velocity of that reaction. In this case, the minimization of the number of loops was a useful strategy to avoid biologically unfeasible values.

### Markov chain Monte Carlo sampling

The fluxes that are possible for each reaction in the model of every strain were obtained using MCMC sampling, as previously described [29]. To model sub-optimal conditions of biomass yield, we used the 90% of experimentally measured growth rate as lower bound of the biomass producing reaction. We used MCMC sampling to obtain two times the number of reactions of the iJO1366 model from the solution space. To gather the samples, we used the gpSampler script of the COBRA Toolbox, which is based on the artificially centered hit-and-run algorithm.

### Enzymatic activity measurements

Each strain at the exponential phase was collected by centrifugation at 5,000 rpm for 10 min. In the case of the NADP^+^-dependent malic enzyme activity, the cells for each strain were resuspended in 500 μL 100 mM KH_2_PO_4_ (pH 7.0), 100 mM NaCl, 2 mM MgCl_2_, 1 mM EDTA, 2 mM dithiothreitol (DTT), and 20% glycerol. For G6PDH, 6PGDH and ICDH activities, cells were resuspended in 500 μL 50 mM Tris-HCl (pH 8.0), 100 mM NaCl and 5 mM MgCl_2_. After sonication for 1 min in an ice bath, the cell debris was removed by centrifugation at 14,000 rpm for 30 min at 4°C. Each enzymatic activity was determined following absorbance at 340 nm in buffer 50 mM Tris-HCl (pH 8.0), 100 mM NaCl, 5 mM MgCl_2_, using the corresponding substrates (malic enzyme: 10 mM L-malate and 0.5 mM NADP^+^; G6PDH: 3.5 mM glucose 6-phosphate and 1.5 mM NADP^+^; 6PGDH: 2.0 mM 6-phosphogluconate and 0.34 mM NADP^+^; ICDH: 1.0 mM isocitrate, 0.34 mM NADP^+^ or 2.0 mM NAD^+^). One unit of specific activity (U × mg^-1^) was defined as 1 μmol NAD(P)^+^ formed per min per mg of protein.

### Statistical analysis

One-way ANOVA and Tukey’s HSD test were performed to analyze all possible pairwise comparisons of each physiological parameter of the strains. A web-application was used to generate automatically a table containing superscript letters that indicate significant differences [30].

## Results and discussion

### The cofactor swapping of ICDH decreases the biomass yield of *E*. *coli* on acetate

In order to evaluate in *E*. *coli* the metabolic impact of changing the cofactor produced by ICDH from NADPH to NADH in the presence and absence of the alternative NADPH source PntAB, we generated the strains *icd*^*NAD*^, Δ*pntAB*, and the double mutant *icd*^*NAD*^ Δ*pntAB*. The genetic modifications were confirmed by sequencing the corresponding loci. The physiological parameters growth rate (μ), acetate uptake rate (Q_ac_) and biomass yield (Y_X/S_) were obtained for these strains at the exponential phase of growth in batch cultures, with acetate as the sole carbon source. When analyzing the HPLC profiles, we did not detect the accumulation of possible by-products. Table 1 shows the obtained values and their pairwise statistical comparisons after performing one-way ANOVA and Tukey’s HSD test.

**Table 1:** Physiological parameters obtained during the exponential growth phase on acetate.

Strain	μ (h^-1^)	Q_ac_ (mmol × gDW^-1^ × h^-1^)	Y_X/S_ (gDW per mmol of acetate)
*wild type*	0.196 ± 0.003 ^a^	7.88 ± 0.02 ^a^	0.0249 ± 0.0004 ^a^
*ΔpntAB*	0.191 ± 0.003 ^a^	7.6 ± 0.2 ^a, b^	0.0250 ± 0.0004 ^a^
*icd*^*NAD*^	0.135 ± 0.004 ^b^	8.0 ± 0.2 ^a^	0.0168 ± 0.0006 ^b^
*icd*^*NAD*^ *ΔpntAB*	0.1203 ± 0.0001 ^c^	7.0 ± 0.2 ^b^	0.0171 ± 0.0004 ^b^

The growth rate (μ), acetate uptake rate (Q_ac_) and biomass yield (Y_X/S_) are shown for each strain used in this work. The mean and the standard error of the mean (SEM) for each physiological parameter were calculated from three different batch cultures. Means without a common superscript letter in the same column differ (P<0.05) as analyzed by one-way ANOVA and the Tukey’s HSD test.

The cofactor swapping of ICDH mainly affected μ and Y_X/S_. These values decreased by a third, irrespective of the presence of PntAB. The lower efficiency in using acetate for biomass production helps to explain why the *icd*^*NAD*^ strain competes worse than wild type on this carbon source, as reported by Zhu *et al*. [3]. Conversely, the absence of PntAB led to no substantial changes in μ and Y_X/S_ when comparing strains with the same cofactor specificity of ICDH. Accordingly, Sauer *et al*. [5] reported the same μ (0.2 h^-1^) for wild type and Δ*pntAB* strains as we observed in batch cultures on acetate (S1 Table). Regarding Q_ac_, neither the cofactor swapping nor the absence of PntAB led to significant differences with the value observed in the wild type strain. Only the double mutant showed around 10% reduction in Q_ac_, when compared to wild type and *icd*^*NAD*^ strains. Furthermore, the double mutant grew at the lowest rate among all the strains.

### Modeling the metabolic effect of cofactor swapping of ICDH

We initially tested our simulations on the capacity of representing the well-known feature that the glyoxylate bypass is essential for the growth of *E*. *coli* on acetate [8]. By using the iJO1366 model, preliminary simulations indicated that *E*. *coli* was able to grow even at zero flux of the reaction catalyzed by ICL. This spurious result was due to pyruvate:ferredoxin oxidoreductase (POR5) performing carbon fixation from CO_2_ (reverse direction of the reaction). When the reaction POR5 was made irreversible by setting up to zero its lower bound, we observed zero production of biomass when there is no flux through ICL (S1 Fig).

Because assuming a total cofactor swapping of ICDH is an important constraint to represent the *in vivo* situation by FBA, we evaluated to what extent the modeling of fluxes depends on that assumption. The physiological parameters obtained for the *icd*^*NAD*^ strain (Table 1) were used as constraints. We examined the objective function (ATPM), the ICDH flux (S2 Fig) and the distribution of fluxes in the central metabolism (S2 Table) as a function of the percentage of change in the reduced cofactor produced (from NADPH to NADH) by the ICDH reaction. Between 0 and 70% of change we observed that the ATPM flux does not respond to the cofactor produced by ICDH; however, between 70% and 100% of change this flux slightly decreased (4%). This subtle decrement was concomitant with even slighter changes in the fluxes of reactions in the glyoxylate bypass and the TCA cycle (S2 Table), such as the flux through ICDH, which changed in 0.3%. Only the transhydrogenase reaction of PntAB responded considerably to the percentage of change in the cofactor produced by ICDH. Thus, the modeled distribution of fluxes in the central pathways relies more on the experimentally measured μ and Q_ac_ of the strain than on the percentage of the reduced cofactor modeled for ICDH. Regarding the cofactor swapping, the kinetic parameters of NAD^+^-dependent ICDH [31] and the concentration of metabolites in *E*. *coli* growing on acetate [32, 33] support an exclusive use of NAD^+^ by this enzyme *in vivo*. Then, even if a little fraction of NADPH is leaking from ICDH in the *icd*^NAD^ strain, we do not expect that the flux distributions *in vivo* will be completely different from the simulations assuming a complete cofactor swapping.

**Fig 1:**
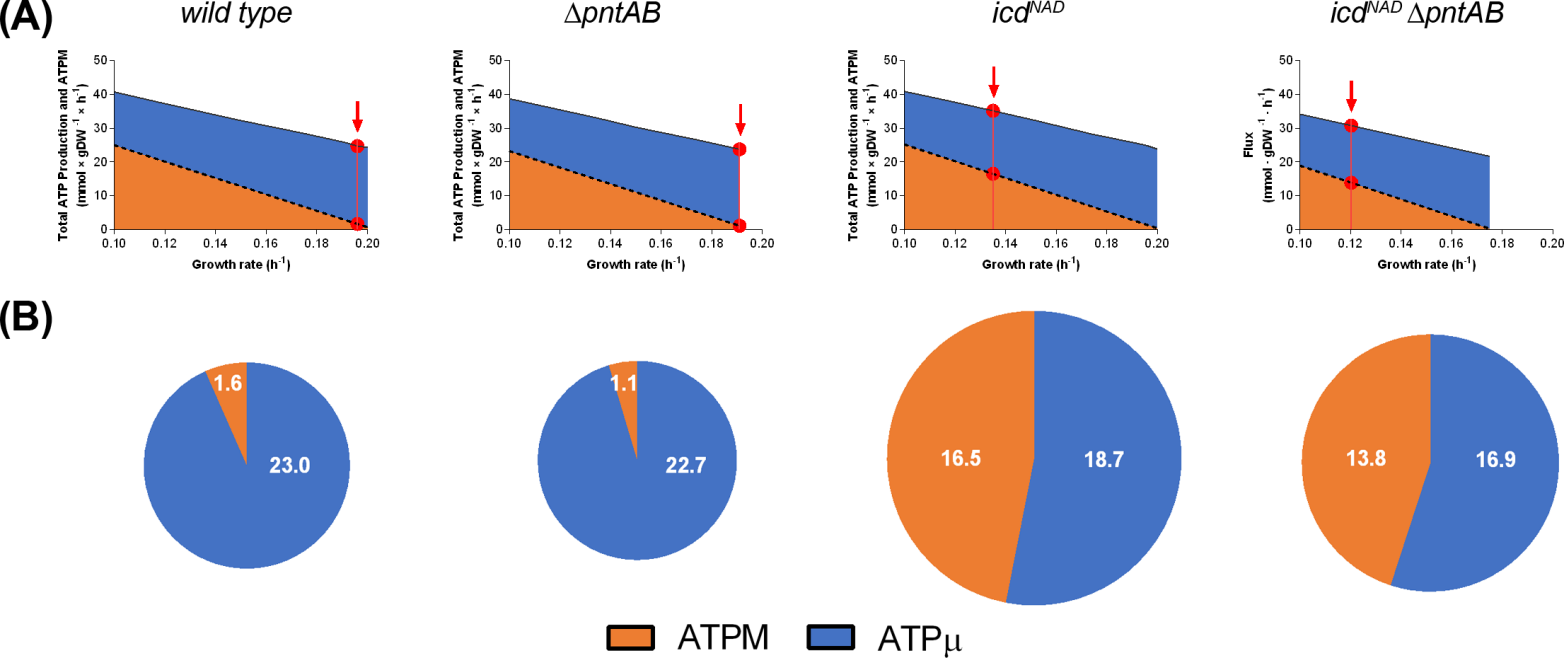
Potential of the studied strains to produce ATP. (A) Robustness analysis to evaluate the maximal flux of the ATPM reaction and the maximal ATP production at different growth rates. The growth rates were varied from 0.1 to 0.2 h^-1^. The red lines and arrows indicate the values obtained for the experimentally measured growth rate. The red circles resulted from the interpolation with the simulated values. For these analyses we used the experimental acetate uptake rate of each strain. (B). Allocation of the total ATP production (mmol × gDW^-1^ × h^-1^) after constraining the respective models with the physiological parameters measured for each strain. The ATP production is shown in two portions: The non-growth associated maintenance requirement (ATPM) and the growth-associated ATP (ATPμ). The latter includes the growth associated maintenance requirement and the ATP used by some pathways (e.g. gluconeogenesis). Fluxes in A and B were calculated from optimal flux distributions of each strain by using the iterative optimization method.

After choosing the maximal production of energy as the optimization criterion to model the metabolic fluxes, which corresponds to maximizing the ATPM flux, we evaluated this reaction at different growth rates for each strain. Fig 1A shows that when the growth rate increases, both the ATPM and the total ATP production decrease, although the first with a higher slope. Conversely, when comparing the different strains, we observed that the responses of ATPM and total ATP production were parallel. In this case, the experimentally measured acetate consumption rate determined the height of the response, *i*.*e*. the double mutant showed the lowest values of ATPM and total ATP production. There is certainly a trade-off between growth rate and energy production yield as previously described [27]. Moreover, when locating the experimental growth rates, it was clear that the strains with lower production of biomass have greater flux through ATPM and lower consumption of ATP for growing purposes (arrows in Fig 1A). In fact, the *icd*^*NAD*^ strains showed at least 10 times greater ATPM flux than the wild type and Δ*pntAB* (Fig 1B). Since the ATPM reaction represents a virtual hydrolysis of ATP, its flux describes the non-growth-associated maintenance requirement. As stated by Pirt [34], the maintenance energy requirement is related to physiological parameters such as μ and Q_ac_, which in this work were used as constraints. Noteworthy, the value obtained for wild type in our simulation is close in magnitude to the value experimentally determined by Farmer and Jones [35] for wild type *E*. *coli* W growing on acetate (3.46 mmol of ATP × gDW^-1^ × h^-1^). Even more, upon cofactor swapping almost half of the production was allocated to non-growth associated maintenance, suggesting the idea that the presence of an NADP^+^-dependent ICDH allows a more efficient use of energy for biomass production when *E*. *coli* grows on acetate.

### Redox balancing response of alternative NADPH sources to the cofactor swapping of ICDH

**Fig 2:**
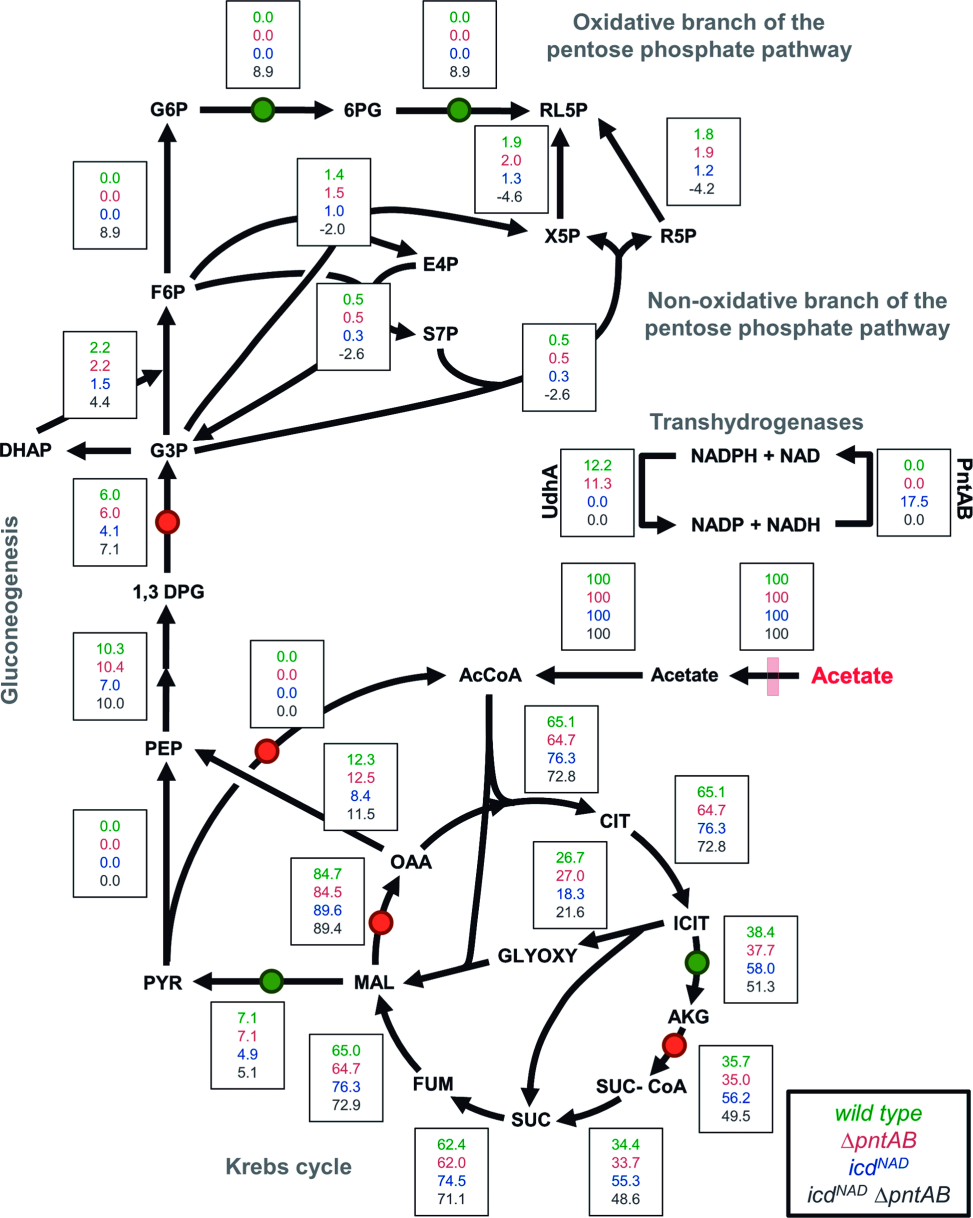
Optimal flux distribution obtained *in silico* for the studied strains. Flux distribution simulated for the acetate metabolism of *E*. *coli* using the FBA-based iterative optimization. The central pathways and the transhydrogenases (UdhA and PntAB) are shown. For each reaction (represented by an arrow), a box containing four values of flux is shown, corresponding to the velocity of reactions simulated in the respective strain (*wild type*, *ΔpntAB*, *icd*^*NAD*^ and *icd*^*NAD*^ *ΔpntAB*). The fluxes are represented as a percentage of the corresponding acetate uptake rate. If the direction of a reaction is opposed to the arrow, the value of flux possesses a negative sign. Green and red circles indicate NADP^+^- and NAD^+^-dependent dehydrogenases, respectively. According to the simulations, only the active malic enzyme (NADP^+^-dependent) is shown. For the FVA flux ranges, which reveal the flexibility of this network, see S3 Table (FVA results).

Optimal flux distributions revealed that the cofactor swapping of ICDH might alter the fluxes of the soluble and membrane-bound transhydrogenases (Fig 2). UdhA was active only in the strains bearing the NADP^+^-dependent ICDH, whereas the flux through PntAB occurred in the *icd*^*NAD*^ strain, but not in the wild type. Accordingly, FVA showed that the maximal flux through UdhA was zero for *icd*^*NAD*^ strains, while a non-zero flux through PntAB was observed only for the *icd*^*NAD*^ strain (S3 Table). These predictions are consistent with previous observations concerning the activity of UdhA in metabolic conditions under excess of NADPH formation [5]. In fact, it has been experimentally determined for wild type *E*. *coli* on acetate that the central pathways produce an excess of ~ 2 mmol × gDW^-1^ × h^-1^ of NADPH over the amount required for growth [33]; very close to our simulated flux of 0.96 mmol × gDW^-1^ × h^-1^ for UdhA. Opposed to the strains bearing an NADP^+^-dependent ICDH, the absence of flux through UdhA in *icd*^*NAD*^ strains suggests that they do not overproduce NADPH when growing on acetate. Even more, the flux through PntAB in the *icd*^*NAD*^ strain suggests that the production of NADPH by the central pathways is insufficient for maximum biomass production when ICDH generates NADH instead of NADPH.

Our simulations showed that in the absence of PntAB, the oxPPP may respond to the cofactor swapping of ICDH (Fig 2). Actually, FVA predicted non-zero minimal and maximal fluxes for the oxPPP in the *icd*^*NAD*^ *ΔpntAB* strain (S3 Table), suggesting that this pathway is essential for that genetic background. However, the production of NADPH by the oxPPP has been reported for wild type *E*. *coli* growing on acetate [36]. Considering that, we performed a sensitivity analysis that showed that the NADP^+^-dependent malic enzyme could replace oxPPP in the production of NADPH, without significantly affecting the biomass yield (S3 Fig). Moreover, FVA showed that only the NADP^+^-dependent malic enzyme should be active in *icd*^*NAD*^ strains, whereas the flux could go through both the NAD^+^- and NADP^+^-dependent malic enzymes in the strains bearing the native ICDH (S3 Table). Interestingly, it has been reported that the deletion of either of the malic enzymes causes identical effects on the growth rate of *E*. *coli* on acetate [37], suggesting an interchangeable role in providing carbon flux towards gluconeogenesis, more than supplying a particular reduced cofactor.

**Fig 3:**
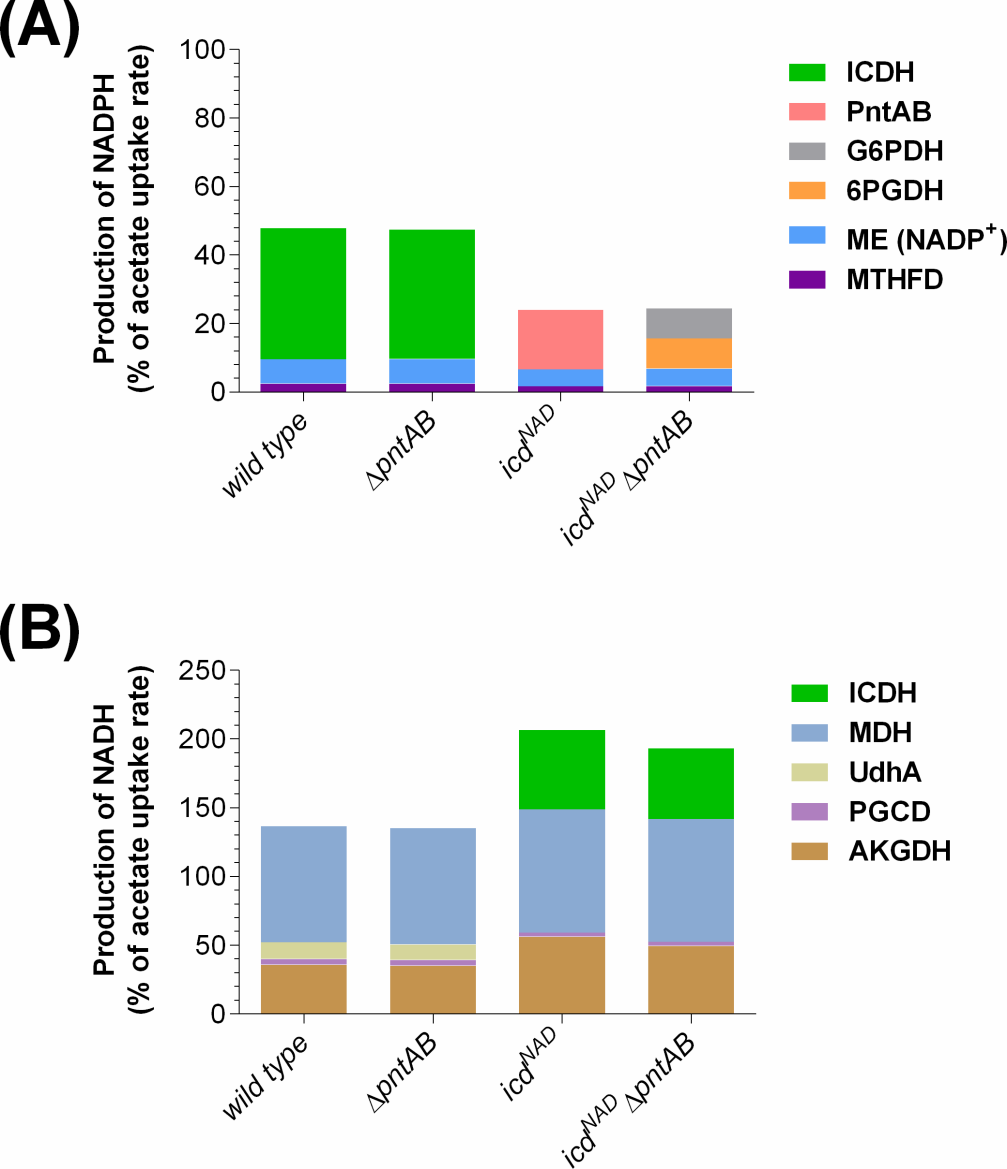
Contribution of different sources to the total production of NADPH and NADH in the studied strains. The flux of production of (a) NADPH and (b) NADH is shown as a percentage of the corresponding acetate uptake rate. The reactions that contribute to the respective cofactor production are detailed. The values were obtained from the optimal flux distribution simulated for each strain used in this work. ICDH: isocitrate dehydrogenase; PntAB: membrane-bound transhydrogenase; G6PDH: glucose 6-phosphate dehydrogenase; 6PGDH: 6-phosphogluconate dehydrogenase; ME: malic enzyme; MTHFD: methylenetetrahydrofolate dehydrogenase; MDH: malate dehydrogenase; UdhA: soluble transhydrogenase; PGCD: phosphoglycerate dehydrogenase; AKGDH: α-ketogluconate dehydrogenase.

When considering the joint contribution of the main sources, we observed that the total production rate of NADPH was similar in the strains bearing the NADP^+^-dependent ICDH, but it decreased by half in the *icd*^*NAD*^ strains (Fig 3A). A diminished NADPH availability is coherent with the decrease in growth rate and biomass yield observed in those strains. Notwithstanding their differences, all strains possessed two common sources of NADPH production: the NADP^+^-dependent malic enzyme and methylenetetrahydrofolate dehydrogenase (MTHFD). Noteworthy, it has been reported that the last enzyme plays an important role in the production of NADPH in eukaryotic cells [38]. As mentioned, the main responses upon cofactor swapping came from PntAB in the *icd*^*NAD*^ strain, and the dehydrogenases of the oxPPP in the double mutant.

**Fig 4:**
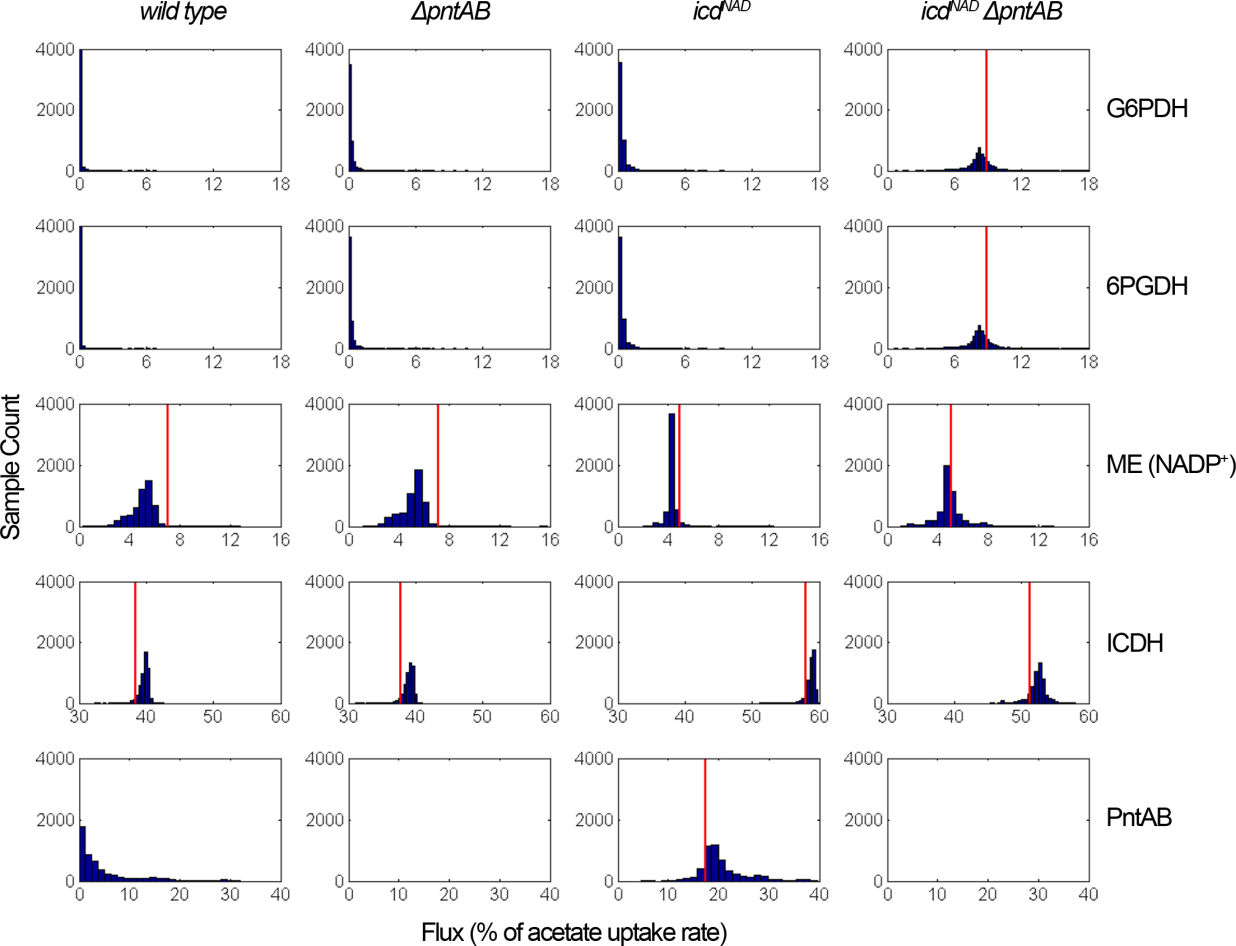
Histograms of sampled fluxes of the main sources of NADPH. Distribution of sampled points from the solution space of each strain under sub-optimal conditions of growth. The histograms of the main sources of NADPH are shown (G6PDH: glucose 6-phosphate dehydrogenase; 6PGDH: 6-phosphogluconate dehydrogenase; ME: malic enzyme; ICDH: isocitrate dehydrogenase; PntAB: membrane-bound transhydrogenase). Simulations were performed using the physiological parameters and a 90% of each growth rate to model the sub-optimal conditions. Also, if the optimal flux was not zero, vertical red lines indicate this value.

Considering that FBA retrieves only the optimal flux distribution, we also explored sub-optimal distributions of fluxes by using Markov chain Monte Carlo sampling (Fig 4). For the main sources of NADPH, the most likely fluxes (the mode of histograms) are quite close to their respective optimal values, however different combinations of fluxes are also possible. In the case of oxPPP dehydrogenases, all strains may sustain with different probabilities the same range of fluxes, although the double mutant favors higher fluxes than the rest of strains. Likewise, for the NADP^+^-dependent malic enzyme the range of possible fluxes is similar for every strain, although the most probable fluxes seemed to be insensitive to the cofactor swapping of ICDH. PntAB covers a wide range of possible fluxes, but the mode of the histogram increased with the change in the reduced cofactor produced by ICDH. In the case of ICDH, histograms showed that the cofactor swapping displaced the mode of the histograms towards considerably higher values, implying an increment in the fraction of acetate converted to CO_2_. In the case of the double mutant, the system required more carbon converted to intermediates in order to generate NADPH through oxPPP, hence the mode was less increased.

Regarding the joint contribution of the main NADH sources, similar trends were observed: strains with identical ICDH did not show differences due to the absence of PntAB, and some of the sources were common for all strains (Fig 3B). After cofactor swapping, the most appreciable changes were the deactivation of UdhA (as described above) and the contribution of ICDH to NADH production. In this situation, the *icd*^*NAD*^ strains showed a significant increment in the total production of NADH and a slight increase of flux through α-ketoglutarate dehydrogenase (AKGDH) and malate dehydrogenase (MDH). Considering the higher ATP yield in the *icd*^NAD^ strains, due to their low growth rate and the trade-off between biomass and energy production [27] (Fig 1A), it is reasonable to suppose that the excess of NADH in these cells is used for the generation of ATP, which in turn is allocated towards ATPM. In this way, the resulting increased energy for maintenance might be dissipating the observed overproduction of NADH. In this regard, other authors have observed both a greater capacity of NADH production and an increased generation of ATP when *E*. *coli* grows at low rates on glucose [39].

### The flux partitioning at the isocitrate bifurcation is affected in strains bearing the NAD^+^-specific ICDH

The partition of fluxes at the isocitrate branch point defines the amount of carbon lost as CO_2_, and hence that available to perform biosynthesis. Thus, we examined how the optimal partition changed with the physiological parameters determined for each strain. Although for all the strains the flux through ICDH was higher than through ICL, this difference is augmented in the *icd*^*NAD*^ strains. S4 Table shows the range of percentages of partition of flux towards ICDH and ICL, as well as the ratios of their reaction velocities (V_ICDH_ / V_ICL_), based on FVA results (S3 Table). Whereas the *icd*^*NAD*^ strain presented 28% more flux towards the TCA cycle than the wild type, that increment was 21% when comparing the *icd*^*NAD*^ Δ*pntAB* strain to Δ*pntAB*. With an increased flux towards the TCA cycle the loss of carbon atoms through CO_2_ also contributes to explain the decrease in biomass yield observed for *icd*^NAD^ strains (Table 1).

**Fig 5:**
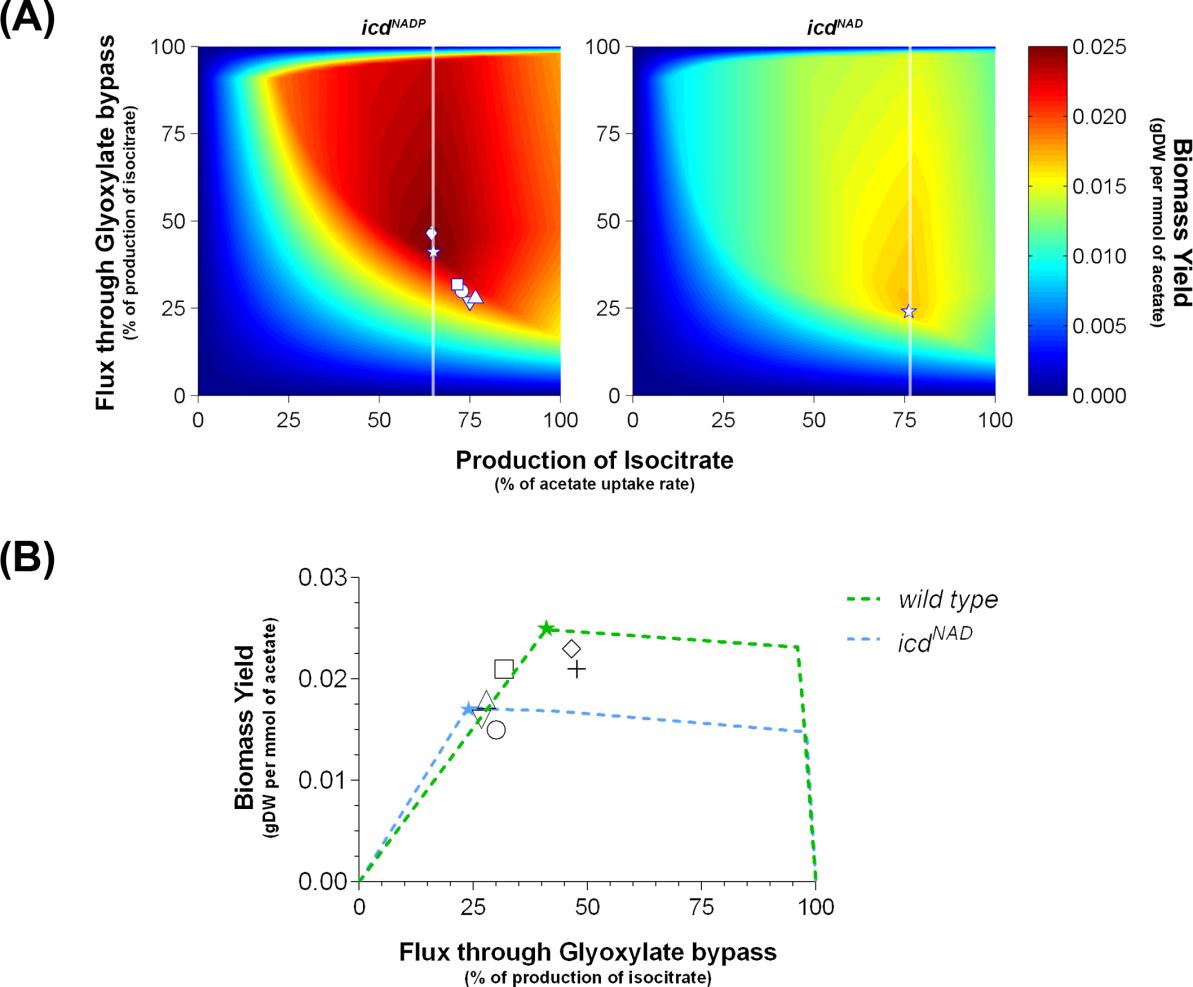
Sensitivity analysis of flux partitioning at the isocitrate bifurcation. (A) Phase plane analysis of biomass yield as a function of the production of isocitrate (x-axis) and the partition of flux through the glyoxylate bypass (y-axis). The analysis was carried out for wild type (labelled as *icd*^*NADP*^) and *icd*^*NAD*^ strains. Experimental data is shown for the wild type strain according to (+) Gerosa *et al*. (2015), (◇) Haverkorn van Rijsewijk (2012), (▽) Taymaz-Nikerel *et al*. (2010), (○) Zhao & Shimizu (2003), (⬜) Holms (1996) and (△) Walsh & Koshland Jr. (1984, 1985). Vertical white lines mark a section of the optimal combinations of production of isocitrate and its partition towards the glyoxylate bypass for the strains under study, which is plotted in (B) as a robustness analysis together with the data obtained from literature.

As a second approach, we studied how the deviation from the optimal partition affected the biomass yield. Fig 5A shows the dependence of biomass yield on both the rate of isocitrate production and the partition of flux towards ICL, simulated by using biomass as the objective function and constraining Q_ac_ and ATPM to the values obtained from wild type or *icd*^*NAD*^ strains. Our analysis shows that the combination of fluxes from both branches of the isocitrate partition is required for growth; i.e. nor 100% of ICDH neither 100% of ICL led to feasible biomass production. This observation coincides with the study of the regulation mechanism of the isocitrate bifurcation in *Mycobacterium* growing on acetate, also obtained by using FBA [40]. At the plane of the optimal rate of isocitrate production, the effect of increasing the partition towards ICL on the biomass yield is described by three regimes (Fig 5B): *i*. a linear increase of the yield until the maximum is reached; *ii*. a slightly declining plateau that extends up to approaching 100% of ICL flux; *iii*. a steep decrease to zero when the partition is fully deviated towards the glyoxylate bypass. Interestingly, the distribution of fluxes experimentally determined in previous reports for wild type *E*. *coli* growing on acetate (S4 Fig) showed that the observed partitions and biomass yields behave similar to our simulations. Indeed, most of the experimental points from previous reports plotted near to the vertex of the optimal partition (41% of flux through glyoxylate bypass, star symbol in Fig 5), mainly within the first regime shown in Fig 5B (differences between experimental and theoretical values are shown in S5 Table). Taking into account our initial analysis constraining the reversibility of POR5 reaction (S1 Fig), the first regime of biomass dependence obeys to the loss of carbon as CO_2_ when flux partitioning favors the TCA cycle branch. We concluded that deviating the partition of flux from the optimum towards ICL has a limited effect on growth, whereas increasing it towards ICDH lead to a fair decrement in the biomass yield.

In the case of simulating with the mutant constraints, the dependence of biomass yield on flux partitioning follows a similar pattern. However, the plateau regime decreased its level, probably as a consequence of constraining with a higher ATPM flux (instead of a possible substrate supply deficiency, given that Q_ac_ of *icd*^*NAD*^ strain is rather similar to wild type), resulting in the establishment of a new optimum value at 24% of partition towards ICL (Fig 5B). The regulation of flux partitioning at the isocitrate bifurcation of *E*. *coli* has been largely attributed to the inactivation of ICDH by phosphorylation [10]. However, it has been demonstrated for the ICDHs from *E*. *coli* and *Saccharomyces cerevisiae* that product inhibition by NADPH renders the enzyme activity sensitive to the redox NADPH/NADP^+^ ratio [41]. Then, one important consequence of cofactor swapping might be the change in this regulation, since the redox ratio NAD^+^/NADH is maintained at a different state within the cell. In this context, an increase in the flux through the NAD^+^-depending ICDH would be expected because of the low concentration of the reduced cofactor relative to the oxidized form. Even more, if such an increase became limiting for the amount of carbon flowing through ICL, a negative regulation of ICDH activity could be needed [10, 12, 13, 42, 43]. Considering the behavior shown by our simulations and the possible responses, we suggest that cofactor specificity of ICDH plays a role in the control of flux partitioning at the isocitrate bifurcation.

### Enzymatic activities of dehydrogenases in cell extracts suggest suboptimal scenarios of flux distribution

We measured the specific activity of NADP^+^-dependent dehydrogenases in cell extracts of each strain as an indicator of particular metabolic responses to ICDH cofactor swapping. However, it is important to keep in mind that the enzymatic activity level is not the only determinant of metabolic fluxes [44]. As shown by the Metabolic Control Analysis framework, other factors could trigger a metabolic response [45]. Our results open more specific questions that should be experimentally evaluated using ^13^C-labeling, metabolomics or transcriptomics in a future work.

**Fig 6:**
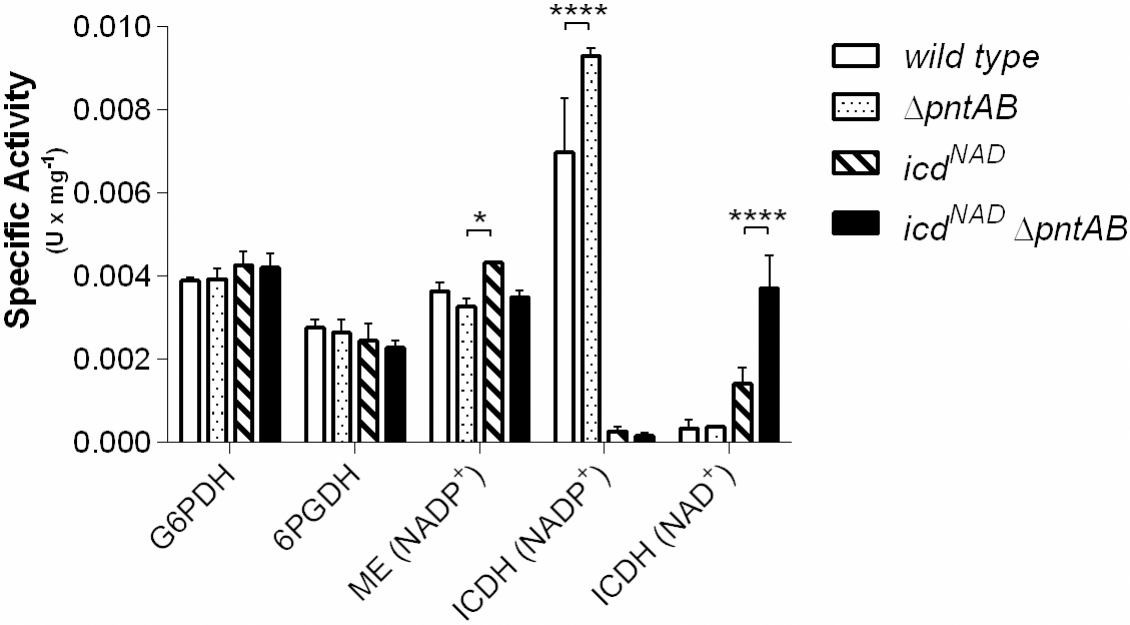
Specific activities of NADPH-producing dehydrogenases of *E*. *coli* during growth on acetate. Activities of the four NADPH-producing dehydrogenases were measured in crude cell extracts for each strain. Cultures were harvested at the exponential phase. Standard deviation is shown for triplicate measurements of each enzyme. ICDH is the only enzyme whose activity was measured in presence of both cofactors, NADP and NAD. G6PDH: glucose 6-phosphate dehydrogenase; 6PGDH: 6-phosphogluconate dehydrogenase; ME: malic enzyme; ICDH: isocitrate dehydrogenase. A p-value < 0.05 is represented by *, while a p-value < 0.0001 is shown as ****.

It is observed in Fig 6 that the specific activities of the dehydrogenases of the oxPPP do not change in any strain. If we assume that this behavior reflects that their metabolic fluxes are indeed similarly active in all strains, then a non-optimal scenario will be taking place (Fig 4). Conversely, the optimal flux distribution predicts that, for the double mutant the production of NADPH is mainly supplied by the oxPPP dehydrogenases, whereas these reactions present zero flux in the other strains (Fig 2). Noteworthy, there are discrepancies about how active is the oxPPP in wild type *E*. *coli* growing on acetate [2, 20, 37, 46–49], while some studies described that the flux is virtually zero, others reported an important flux through this pathway (S4 Fig). If all the strains had a similar basal flux or inactivation of the oxPPP, a change in the use of the malic enzymes would be expected as an alternative response to ICDH cofactor swapping. However, the specific activity of the NADP^+^-dependent malic enzyme is similar among all the strains, in agreement with both optimal and sub-optimal simulations. These results suggest that the NADPH-balancing response may not be operating at an optimal state.

Concerning the specific activity of ICDH, extracts of *icd*^*NAD*^ strains showed lower values than the parental strains (Fig 6), indicating an altered flux partitioning. Thus, whereas the optimal behavior predicts an increment of the flux through ICDH upon cofactor swapping, enzymatic activity pointed towards the opposite case. We cannot discard that a negative regulatory response, perhaps through ICDH phosphorylation, may be causing non-optimal activation of the glyoxylate bypass. In fact, a moderate increased flux through ICL should not greatly affect biomass yield (plateau zone of Fig 5B); however, according to the sampling of suboptimal fluxes (Fig 4) the minimal value of the ICDH flux in the *icd*^NAD^ strain still should be greater than the maximal value observed in wild type. Remarkably, although optimal flux distributions showed that ICDH hardly responded to the presence or absence of PntAB (Figs 2 and 3), we observed an increased ICDH activity in the extracts of *ΔpntAB* strains for both the NAD^+^- and NADP^+^-dependent forms (Fig 6). This behavior could be possible under sub-optimal scenarios (Fig 4), but at low probability. Interestingly, the expression level of PntAB has been correlated with the ratio between α-ketoglutarate and glutamine [50]. In this sense, with the change in cofactor specificity of ICDH, a reduced synthesis of glutamine might be expected (caused by NADPH deficit, since this amino acid is produced from glutamate and NADPH [51]). Then, a concomitant accumulation of α-ketoglutarate (the precursor of glutamate) is also expected, triggering an increased expression of PntAB. Interestingly, activation of the PntAB flux was observed in our simulations (Figs 2 and 4). In this context, a low specific activity of ICDH (Fig 6) may also result from a homeostatic regulation to counter a possible excess of α-ketoglutarate.

If the *icd*^NAD^ strains are not operating at an optimal state, this might be due to regulatory responses to the presence of a NADH-producing ICDH, causing activation of suboptimal pathways. Besides the aforementioned case, we explored the idea of suboptimal pathway activation in regard to NADPH production. To simulate this, we selected sub-optimal flux distributions (from those showed in Fig 4) whose flux through PntAB was lower than its optimal flux in *icd*^*NAD*^ strain, since this enzyme resulted the main optimal response after cofactor swapping (Fig 2). As expected, the probability of optimal fluxes decreased, whereas the probability of higher flux values for oxPPP and malic enzyme increased (S5 Fig). In this condition, the possible fluxes of ICDH are distributed almost homogeneously in a range of ~50–60% of acetate uptake rate.

To conclude, our work was focused on revealing the most important aspects about the metabolic responses most likely to occur after cofactor swapping of ICDH. We found that the cofactor swapping of ICDH led to insufficient production of NADPH from central pathways during growth of *E*. *coli* on acetate. A role of PntAB in balancing the NADPH deficiency is suggested, although its deletion did not cause a significant impact over the physiological parameters. In the absence of PntAB, oxPPP and/or malic enzyme are the suggested NADPH suppliers, in spite of presenting similar specific activities in all genetic contexts. Our experimental results and computational analyses suggest that given the trade-off between the growth rate and total ATP yield, and the limited availability of intermediates for biomass production in the *icd*^*NAD*^ strains, a significant part of the ATP production becomes allocated towards non-growing purposes. Finally, we propose that the cofactor specificity of ICDH affects the control of flux partitioning at the isocitrate bifurcation.

## Supporting information

S1 Fig Impact of constraining the reversibility of pyruvate:Ferredoxin oxidoreductase on simulations. Robustness analysis simulating the impact of a variable flux through the glyoxylate bypass, as a percentage of the total flux divided between ICL and ICDH (the total is equivalent to the net flux of production of isocitrate, which was maintained constant in these simulations), on the specific rate of growth on acetate as sole carbon source. When the reverse direction of the reaction catalyzed by pyruvate:ferredoxin oxidoreductase (POR5) was allowed (without constraint), growth was possible with no flux through the glyoxylate bypass (biomass yield was not zero). However, after correction of reversibility of POR5 (with constraint), avoiding carbon fixation in the reverse direction, there was no growth as experimentally expected [8].
(TIFF)

S2 Fig Effect of different percentages of cofactor swapping of ICDH. Evaluation of the effect that could have a non-complete cofactor swapping of ICDH over the objective function (maximal flux through the ATPM reaction) and the flux through this dehydrogenase. A scanning from 0 to 100% of cofactor swapping was done by using physiological parameters of the *icd*^*NAD*^ strain as constraints. A percentage of 0% of cofactor swapping means that the enzyme uses 0% of NAD^+^ and 100% of NADP^+^, whereas a complete shifting of cofactor specificity represents a use of 100% of NAD^+^ and 0% of NADP^+^.
(TIFF)

S3 Fig Effect of flux through the NADP^+^-dependent malic enzyme and the oxPPP over the biomass yield. Phase plane analysis of biomass yield as a function of the flux through the NADP^+^-dependent malic enzyme (x-axis) and the flux through oxPPP (y-axis). The behavior of each strain is shown, arranged by presence of *icd*^*NADP*^ or *icd*^*NAD*^ gene (columns) and by presence or absence of *pntAB* operon (rows). FBA-calculated optimal values are shown as (☆). Experimental data is shown for the wild type strain: (+) Gerosa *et al*. (2015), (◇) Haverkorn van Rijsewijk (2012), (▽) Taymaz-Nikerel *et al*. (2010), (○) Zhao & Shimizu (2003), (⬜) Holms (1996) and (△) Walsh & Koshland Jr. (1984, 1985).
(TIFF)

S4 Fig Reported flux distributions in wild type *E*. *coli* grown on acetate as sole carbon source. Flux distributions in acetate metabolism of *E*. *coli* reported by different authors, using diverse methods to determine net fluxes. Central pathways are shown. For each reaction (represented by an arrow) a box containing six values of flux is shown, corresponding to the velocity of reaction reported by Gerosa *et al*. (2015), Haverkorn van Rijsewijk (2012), Taymaz-Nikerel *et al*. (2010), Zhao & Shimizu (2003), Holms (1996) and Walsh & Koshland Jr. (1984, 1985), respectively. With the exception of Taymaz-Nikerel *et al*. (2010), all studies used ^13^C isotope labeling to determine flux distributions. The fluxes are represented as a percentage of the corresponding acetate uptake rate. If the direction of a reaction is opposed to that of the arrow, the value of flux possesses a negative sign. Dehydrogenases are represented by green and red circles, indicating NADP^+^ and NAD^+^ specificity, respectively. Only the NADP^+^-dependent malic enzyme is shown. n.d., not determined.
(TIFF)

S5 Fig Histograms of sub-optimal flux distributions after filtering the flux of PntAB. Distribution of selected-sampled points from the solution space of the *icd*^*NAD*^ strain under sub-optimal conditions of growth. In this case we used as upper bound the 75% of the optimal flux of PntAB. The histograms of the main sources of NADPH are shown (G6PDH: glucose 6-phosphate dehydrogenase; 6PGDH: 6-phosphogluconate dehydrogenase; ME: malic enzyme; ICDH: isocitrate dehydrogenase; PntAB: membrane-bound transhydrogenase). If the optimal flux was not zero, vertical red lines indicate this value. The flux distributions selected were gathered from the samples used for Fig 4.
(TIFF)

S1 Table Physiological data from the literature for wild type *E*. *coli* grown on acetate. (DOCX)

S2 Table Optimal flux distributions of central pathways and transhydrogenases under different percentages of cofactor swapping of ICDH. (DOCX)

S3 Table Flux variability analysis of central pathways and transhydrogenases. (DOCX)

S4 Table Partition of flux at the branch point between the Krebs cycle and the glyoxylate bypass. (DOCX)

S5 Table Theoretical biomass yield predicted for wild type *E*. *coli* grown on acetate. (DOCX)
