# Supplementary figures and images for: Understanding the impact of the cofactor swapping of isocitrate dehydrogenase over the growth phenotype of Escherichia coli on acetate by using constraint-based modeling
Source: PLoS One. 2018 Apr 20;13(4):e0196182. doi: 10.1371/journal.pone.0196182 (PMC5909895; doi:10.1371/journal.pone.0196182)

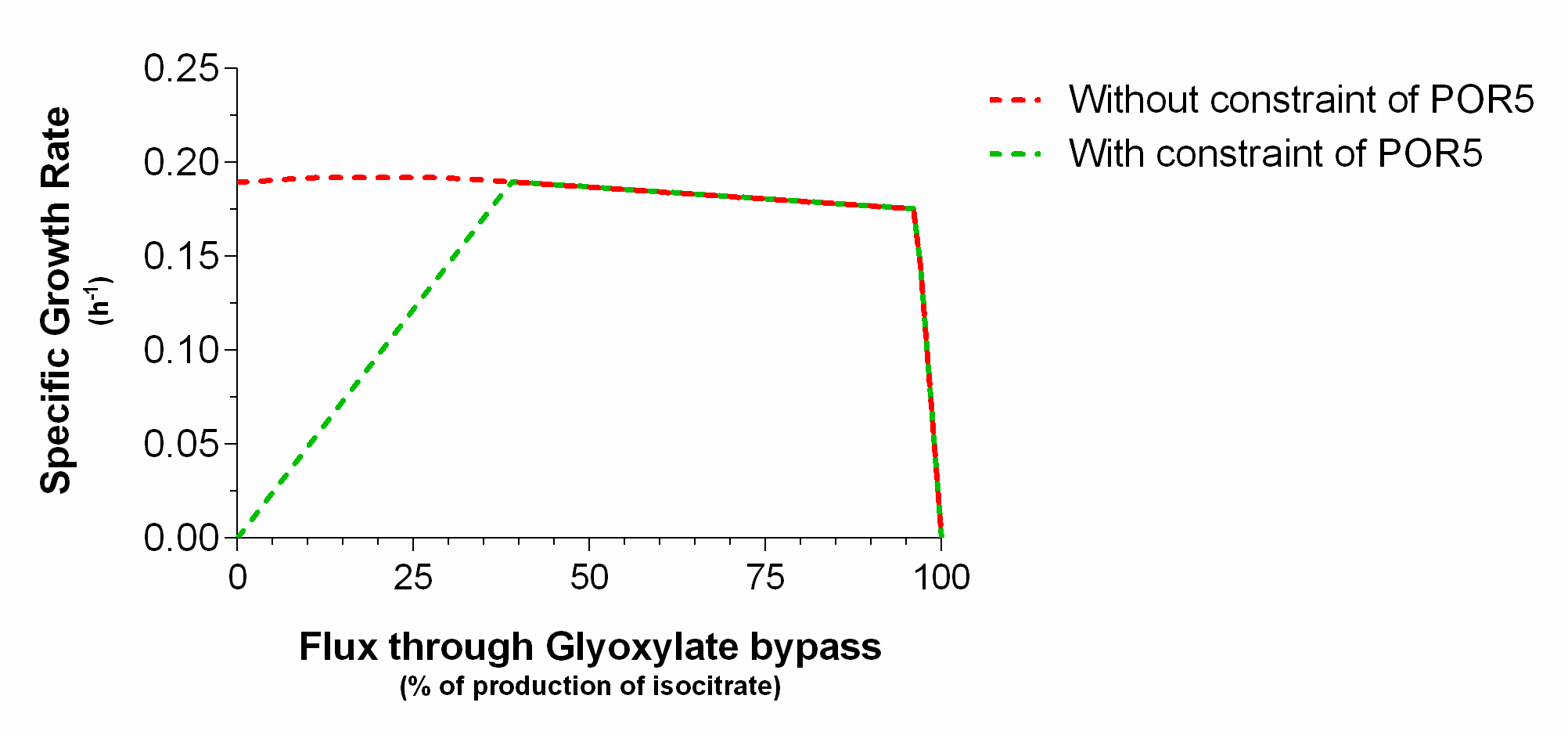

Supplement: S1 Fig — Robustness analysis simulating the impact of a variable flux through the glyoxylate bypass, as a percentage of the total flux divided between ICL and ICDH (the total is equivalent to the net flux of production of isocitrate, which was maintained constant in these simulations), on the specific rate of growth on acetate as sole carbon source. When the reverse direction of the reaction catalyzed by pyruvate:ferredoxin oxidoreductase (POR5) was allowed (without constraint), growth was possible with no flux through the glyoxylate bypass (biomass yield was not zero). However, after correction of reversibility of POR5 (with constraint), avoiding carbon fixation in the reverse direction, there was no growth as experimentally expected [8]. (TIFF) [file pone.0196182.s001.tiff]

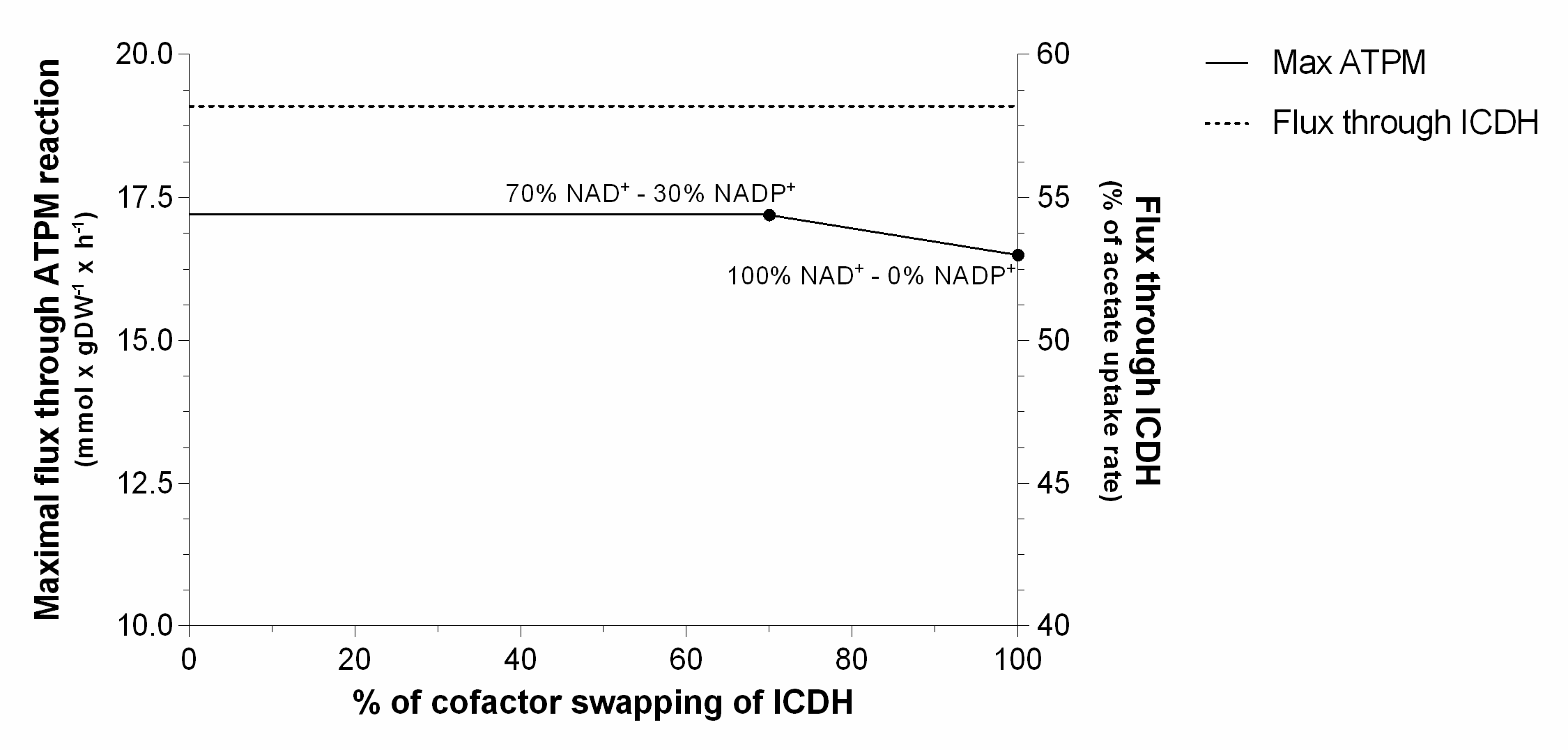

Supplement: S2 Fig — Evaluation of the effect that could have a non-complete cofactor swapping of ICDH over the objective function (maximal flux through the ATPM reaction) and the flux through this dehydrogenase. A scanning from 0 to 100% of cofactor swapping was done by using physiological parameters of the icdNAD strain as constraints. A percentage of 0% of cofactor swapping means that the enzyme uses 0% of NAD+ and 100% of NADP+, whereas a complete shifting of cofactor specificity represents a use of 100% of NAD+ and 0% of NADP+. (TIFF) [file pone.0196182.s002.tiff]

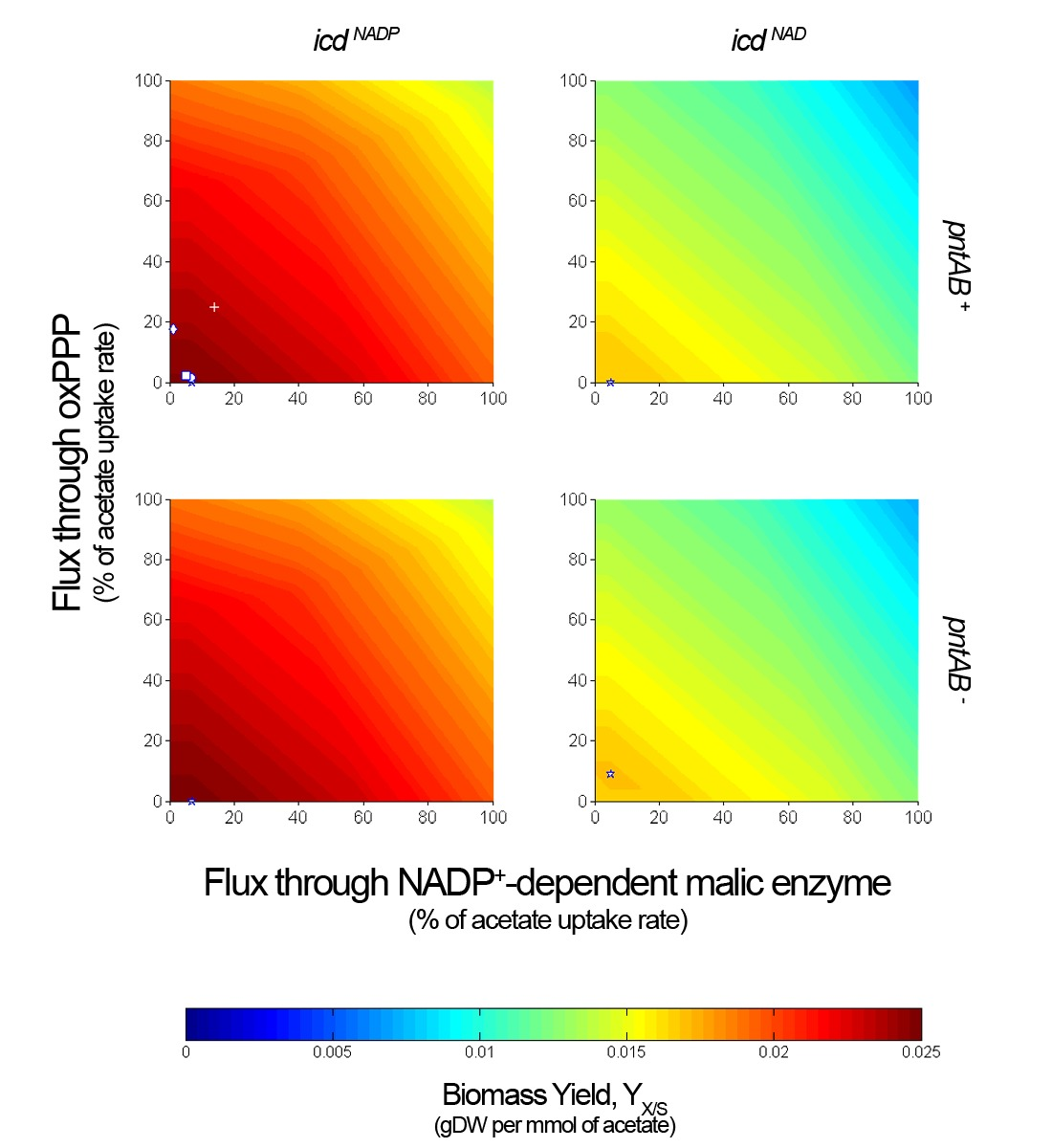

Supplement: S3 Fig — Phase plane analysis of biomass yield as a function of the flux through the NADP+-dependent malic enzyme (x-axis) and the flux through oxPPP (y-axis). The behavior of each strain is shown, arranged by presence of icdNADP or icdNAD gene (columns) and by presence or absence of pntAB operon (rows). FBA-calculated optimal values are shown as (☆). Experimental data is shown for the wild type strain: (+) Gerosa et al. (2015), (◇) Haverkorn van Rijsewijk (2012), (▽) Taymaz-Nikerel et al. (2010), (○) Zhao & Shimizu (2003), (⬜) Holms (1996) and (△) Walsh & Koshland Jr. (1984, 1985). (TIFF) [file pone.0196182.s003.tiff]

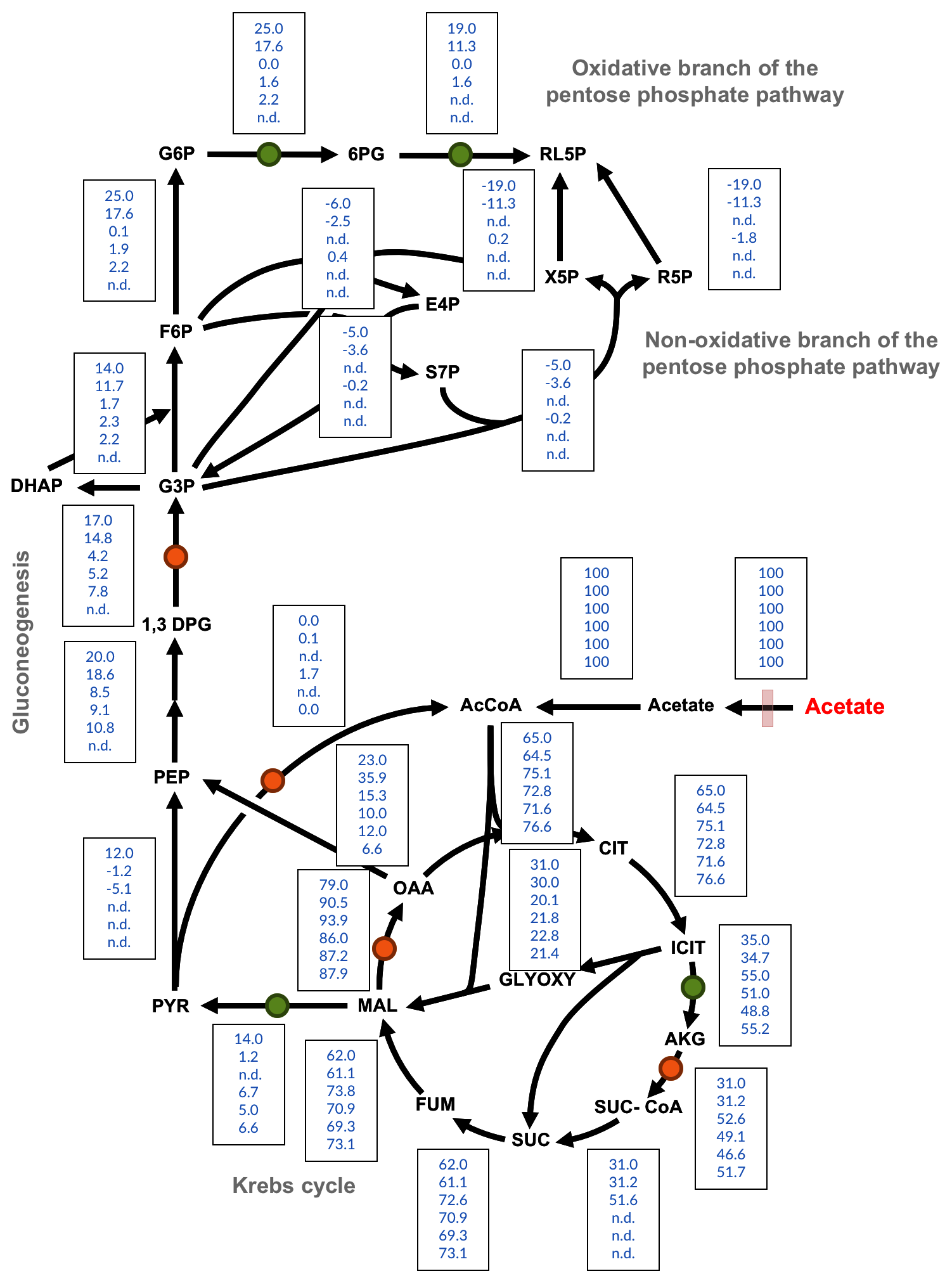

Supplement: S4 Fig — Flux distributions in acetate metabolism of E. coli reported by different authors, using diverse methods to determine net fluxes. Central pathways are shown. For each reaction (represented by an arrow) a box containing six values of flux is shown, corresponding to the velocity of reaction reported by Gerosa et al. (2015), Haverkorn van Rijsewijk (2012), Taymaz-Nikerel et al. (2010), Zhao & Shimizu (2003), Holms (1996) and Walsh & Koshland Jr. (1984, 1985), respectively. With the exception of Taymaz-Nikerel et al. (2010), all studies used 13C isotope labeling to determine flux distributions. The fluxes are represented as a percentage of the corresponding acetate uptake rate. If the direction of a reaction is opposed to that of the arrow, the value of flux possesses a negative sign. Dehydrogenases are represented by green and red circles, indicating NADP+ and NAD+ specificity, respectively. Only the NADP+-dependent malic enzyme is shown. n.d., not determined. (TIFF) [file pone.0196182.s004.tiff]

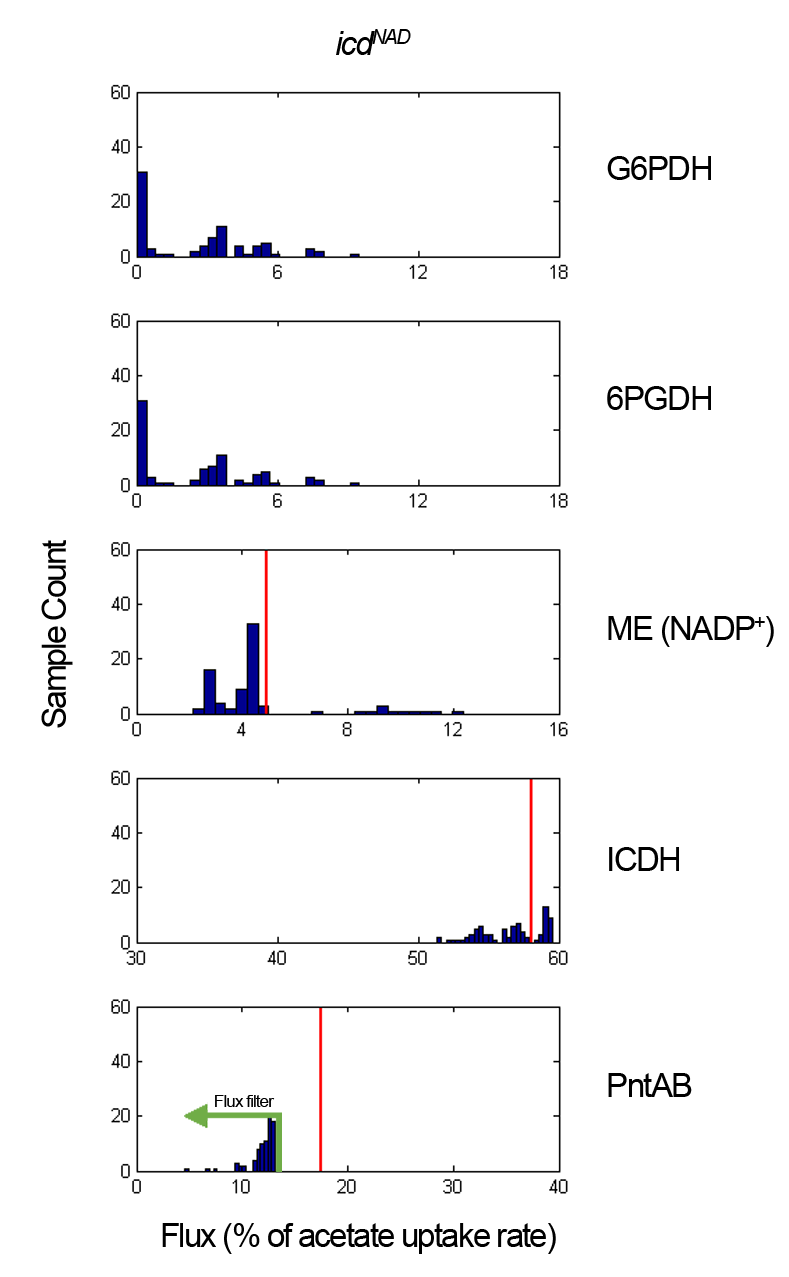

Supplement: S5 Fig — Distribution of selected-sampled points from the solution space of the icdNAD strain under sub-optimal conditions of growth. In this case we used as upper bound the 75% of the optimal flux of PntAB. The histograms of the main sources of NADPH are shown (G6PDH: glucose 6-phosphate dehydrogenase; 6PGDH: 6-phosphogluconate dehydrogenase; ME: malic enzyme; ICDH: isocitrate dehydrogenase; PntAB: membrane-bound transhydrogenase). If the optimal flux was not zero, vertical red lines indicate this value. The flux distributions selected were gathered from the samples used for Fig 4. (TIFF) [file pone.0196182.s005.tiff]
