# Supplementary material for: Understanding the impact of the cofactor swapping of isocitrate dehydrogenase over the growth phenotype of Escherichia coli on acetate by using constraint-based modeling
Source: PLoS One. 2018 Apr 20;13(4):e0196182. doi: 10.1371/journal.pone.0196182 (PMC5909895; doi:10.1371/journal.pone.0196182)
Supplement: S1 Table — (DOCX) [file pone.0196182.s006.docx]

| **Wild type strain** | **μ (h^-1^)** | **Q_ac_ (mmol** **× gDW^-1^** **× h^-1^)** | **Y_X/S_ (gDW per mmol of acetate)** | **Culture medium** | **Reference** |
| --- | --- | --- | --- | --- | --- |
| BW25113 | 0.29 | 12.58 | 0.023 | M9  acetate 5 g × L^-1^ | [33] ^a^ |
| BW25113 | 0.29 | 13.58 | 0.021 | M9  acetate 5 g × L^-1^ | [36] ^a^ |
| K-12 | 0.22 | 14.43 | 0.015 | M9  sodium acetate 2 g × L^-1^ | [2] ^b^ |
| K-12 | 0.11 | 7.18 | 0.015 | M9  sodium acetate 2 g × L^-1^ | [2] ^b^ |
| K-12 | 0.29 | 16.31 | 0.018 | MOPS  acetate 5.9 g × L^-1^ | [48, 49] ^a^ |
| MC4100 | 0.33 | n.d. | n.d. | M9  acetate 2.5 g × L^-1^ | [37] ^a^ |
| MG1655 | 0.31 | 15.5 | 0.020 | M9  acetate 4 g × L^-1^ | [17] ^a^ |
| MG1655 | 0.20 | n.d. | n.d. | M9  acetate 5 g × L^-1^ | [5] ^a^ |
| MG1655 | 0.12 | 7.0 | 0.016 | MOPS  acetate 6.7 g × L^-1^ | [47] ^b^ |
| MG1655 | 0.35 | n.d. | n.d. | MOPS  acetate 20 g × L^-1^ | [7] ^a^ |
| ML308 | 0.43 | 20.16 | 0.021 | LMR  acetate 1.77 g × L^-1^ | [46] ^a^ |

The growth rate (μ), acetate uptake rate (Q_ac_) and biomass yield (Y_X/S_) are shown for each case. Parameters that were not determined are shown as n.d. The gray-filled cells represent the studies where flux distributions were reported.

^a^ : Batch culture.

^b^ : Continuous culture.
