# Supplementary material for: Understanding the impact of the cofactor swapping of isocitrate dehydrogenase over the growth phenotype of Escherichia coli on acetate by using constraint-based modeling
Source: PLoS One. 2018 Apr 20;13(4):e0196182. doi: 10.1371/journal.pone.0196182 (PMC5909895; doi:10.1371/journal.pone.0196182)
Supplement: S2 Table — (DOCX) [file pone.0196182.s007.docx]

|  | ***icd^NAD^*** | |
| --- | --- | --- |
| **Rxn Name** | **70% NAD - 30% NADP** | **100% NAD - 0% NADP** |
| **G6PDH2r** | 0.0 | 0.0 |
| **PGL** | 0.0 | 0.0 |
| **GND** | 0.0 | 0.0 |
| **RPE** | -1.3 | -1.3 |
| **RPI** | -1.2 | -1.2 |
| **TKT1** | -0.3 | -0.3 |
| **TKT2** | -1.0 | -1.0 |
| **TALA** | -0.3 | -0.3 |
| **PGI** | 0.0 | 0.0 |
| **FBP** | 1.5 | 1.5 |
| **PFK** | 0.0 | 0.0 |
| **FBA** | -1.5 | -1.5 |
| **GAPD** | -4.1 | -4.1 |
| **PGK** | 4.1 | 4.1 |
| **PGM** | 7.0 | 7.0 |
| **ENO** | -7.0 | -7.0 |
| **PPS** | 0.0 | 0.0 |
| **PYK** | 0.0 | 0.0 |
| **PFL** | 0.0 | 0.0 |
| **PDH** | 0.0 | 0.0 |
| **NADTRHD** | 0.0 | 0.0 |
| **THD2pp** | 0.3 | 17.5 |
| **PPC** | 0.0 | 0.0 |
| **PPCK** | 8.4 | 8.4 |
| **CS** | 76.3 | 76.3 |
| **ACONTa** | 76.3 | 76.3 |
| **ACONTb** | 76.3 | 76.3 |
| **ICDHyr** | 58.2 | 58.0 |
| **AKGDH** | 56.4 | 56.2 |
| **SUCOAS** | -55.5 | -55.3 |
| **SUCDi** | 254.2 * | 74.5 |
| **FUM** | 76.3 | 76.3 |
| **MDH** | 89.6 | 89.6 |
| **ME1** | 0.0 | 0.0 |
| **ME2** | 4.8 | 4.9 |
| **ICL** | 18.1 | 18.3 |
| **MALS** | 18.1 | 18.3 |

The flux values are represented as a percentage of the corresponding acetate uptake rate. These flux distributions were obtained by using the iterative optimization method after constraining the model with the respective physiological parameters of *icd^NAD^* strain.

^a^: The names of each reaction are based on the nomenclature used in iJO1366 model of *E. coli.*

*: This value is because of a loop in the flux distribution.
