# Supplementary material for: Understanding the impact of the cofactor swapping of isocitrate dehydrogenase over the growth phenotype of Escherichia coli on acetate by using constraint-based modeling
Source: PLoS One. 2018 Apr 20;13(4):e0196182. doi: 10.1371/journal.pone.0196182 (PMC5909895; doi:10.1371/journal.pone.0196182)
Supplement: S3 Table — (DOCX) [file pone.0196182.s008.docx]

|  | ***wild type*** | | ***ΔpntAB*** | | ***icd^NAD^*** | | ***icd^NAD^ ΔpntAB*** | |
| --- | --- | --- | --- | --- | --- | --- | --- | --- |
| **Rxn Name ^a^** | **Min** | **Max** | **Min** | **Max** | **Min** | **Max** | **Min** | **Max** |
| **G6PDH2r** | 0.0 | 0.0 | 0.0 | 0.0 | 0.0 | 0.0 | 8.9 | 8.9 |
| **PGL** | 0.0 | 0.0 | 0.0 | 0.0 | 0.0 | 0.0 | 8.9 | 8.9 |
| **GND** | 0.0 | 0.0 | 0.0 | 0.0 | 0.0 | 0.0 | 8.9 | 8.9 |
| **RPE** | -1.9 | -1.9 | -2.0 | -2.0 | -1.3 | -1.3 | 4.6 | 4.6 |
| **RPI** | -1.8 | -1.8 | -1.9 | -1.9 | -1.2 | -1.2 | -4.2 | -4.2 |
| **TKT1** | -0.5 | -0.5 | -0.5 | -0.5 | -0.3 | -0.3 | 2.6 | 2.6 |
| **TKT2** | -1.4 | -1.4 | -1.5 | -1.5 | -1.0 | -1.0 | 2.0 | 2.0 |
| **TALA** | -0.5 | -0.5 | -0.5 | -0.5 | -0.3 | -0.3 | 2.6 | 2.6 |
| **PGI** | 0.0 | 0.0 | 0.0 | 0.0 | 0.0 | 0.0 | -8.9 | -8.9 |
| **FBP** | 0.0 | 2.2 | 0.0 | 2.2 | 1.5 | 1.5 | 4.4 | 4.4 |
| **PFK** | 0.0 | 0.0 | 0.0 | 0.0 | 0.0 | 0.0 | 0.0 | 0.0 |
| **FBA** | -2.2 | 0.0 | -2.2 | 0.0 | -1.5 | -1.5 | -4.4 | -4.4 |
| **GAPD** | -6.0 | -6.0 | -6.0 | -6.0 | -4.1 | -4.1 | -7.1 | -7.1 |
| **PGK** | 6.0 | 6.0 | 6.0 | 6.0 | 4.1 | 4.1 | 7.1 | 7.1 |
| **PGM** | 10.3 | 10.3 | 10.4 | 10.4 | 7.0 | 7.0 | 10.0 | 10.0 |
| **ENO** | -10.3 | -10.3 | -10.4 | -10.4 | -7.0 | -7.0 | -10.0 | -10.0 |
| **PPS** | 0.0 | 0.0 | 0.0 | 0.0 | 0.0 | 0.0 | 0.0 | 0.0 |
| **PYK** | 0.0 | 7.1 | 0.0 | 7.1 | 0.0 | 0.0 | 0.0 | 0.0 |
| **PFL** | 0.0 | 0.0 | 0.0 | 0.0 | 0.0 | 0.0 | 0.0 | 0.0 |
| **PDH** | 0.0 | 0.0 | 0.0 | 0.0 | 0.0 | 0.0 | 0.0 | 0.0 |
| **NADTRHD** | 0.0 | 12.2 | 0.0 | 11.3 | 0.0 | 0.0 | 0.0 | 0.0 |
| **THD2pp** | 0.0 | 0.0 | 0.0 | 0.0 | 17.4 | 17.8 | 0.0 | 0.0 |
| **PPC** | 0.0 | 0.0 | 0.0 | 0.0 | 0.0 | 0.0 | 0.0 | 0.0 |
| **PPCK** | 12.3 | 19.4 | 12.5 | 19.6 | 8.4 | 8.4 | 11.5 | 11.5 |
| **CS** | 65.1 | 65.1 | 64.7 | 64.7 | 76.3 | 76.3 | 72.9 | 72.9 |
| **ACONTa** | 65.1 | 65.1 | 64.7 | 64.7 | 76.3 | 76.3 | 72.9 | 72.9 |
| **ACONTb** | 65.1 | 65.1 | 64.7 | 64.7 | 76.3 | 76.3 | 72.9 | 72.9 |
| **ICDHyr** | 38.4 | 38.4 | 37.7 | 37.7 | 58.0 | 58.2 | 51.3 | 51.3 |
| **AKGDH** | 35.7 | 35.7 | 35.0 | 35.0 | 56.2 | 56.4 | 49.5 | 49.5 |
| **SUCOAS** | u.v. | -34.4 | u.v. | -33.7 | u.v. | -55.3 | u.v. | -48.6 |
| **SUCDi** | 62.4 | 185.2 | 62.0 | 182.9 | 74.5 | 254.2 | 71.1 | 251.6 |
| **FUM** | 65.0 | 77.2 | 64.7 | 75.9 | 76.3 | 76.3 | 72.9 | 72.9 |
| **MDH** | 84.7 | 96.9 | 84.5 | 95.8 | 89.6 | 89.6 | 89.4 | 89.4 |
| **ME1** | 0.0 | 7.1 | 0.0 | 7.1 | 0.0 | 0.0 | 0.0 | 0.0 |
| **ME2** | 0.0 | 7.1 | 0.0 | 7.1 | 4.8 | 5.0 | 5.1 | 5.1 |
| **ICL** | 26.7 | 26.7 | 27.0 | 27.0 | 18.1 | 18.3 | 21.6 | 21.6 |
| **MALS** | 26.7 | 26.7 | 27.0 | 27.0 | 18.1 | 18.3 | 21.6 | 21.6 |

The flux values are represented as a percentage of the corresponding acetate uptake rate. The gray-filled rows represent the relevant reactions for this work.

^a^: The names of each reaction are based on the nomenclature used in iJO1366 model of *E. coli.*

u.v.: unrepresentative value (percentage calculated from a flux value of ±1000 mmol × gDW^-1^ × h^-1^).
