# Supplementary material for: Understanding the impact of the cofactor swapping of isocitrate dehydrogenase over the growth phenotype of Escherichia coli on acetate by using constraint-based modeling
Source: PLoS One. 2018 Apr 20;13(4):e0196182. doi: 10.1371/journal.pone.0196182 (PMC5909895; doi:10.1371/journal.pone.0196182)
Supplement: S4 Table — (DOCX) [file pone.0196182.s009.docx]

| **Strain** | **Partition of flux to**  **ICDH (Krebs cycle)** | **Partition of flux to**  **ICL (Glyoxylate bypass)** | **Ratio**  **V_ICDH_ / V_ICL_** |
| --- | --- | --- | --- |
| wild type | 59% | 41% | 1.44 |
| *ΔpntAB* | 58% | 42% | 1.38 |
| *icd^NAD^* | 76 – 76.3% | 23.7 – 24% | 3.17 – 3.22 |
| *icd^NAD^ ΔpntAB* | 70% | 30% | 2.33 |

The respective values of partition are a percentage of the net flux of conversion from citrate to isocitrate in each case. The results were obtained from the flux variability analysis detailed on S3 Table.
