# Supplementary material for: Understanding the impact of the cofactor swapping of isocitrate dehydrogenase over the growth phenotype of Escherichia coli on acetate by using constraint-based modeling
Source: PLoS One. 2018 Apr 20;13(4):e0196182. doi: 10.1371/journal.pone.0196182 (PMC5909895; doi:10.1371/journal.pone.0196182)
Supplement: S5 Table — (DOCX) [file pone.0196182.s010.docx]

| **Reference** | **μ (h^-1^)** | **Q_ac_ (mmol** **× gDW^-1^** **× h^-1^)** | **Y_X/S_ (gDW per mmol of acetate)** | **Theoretical Y_X/S_ (gDW per mmol of acetate) ^c^** | **Difference between experimental and theoretical data (%)** |
| --- | --- | --- | --- | --- | --- |
| Gerosa *et al.* (2015) [36] ^a^ | 0.29 | 13.58 | 0.021 | 0.025 | 16 |
| Haverkorn (2012) [33] ^a^ | 0.29 | 12.58 | 0.023 | 0.025 | 7 |
| Taymaz-Nikerel *et al*. (2010) [47] ^b^ | 0.12 | 7.00 | 0.016 | 0.019 | 15 |
| Zhao & Shimizu (2003) [2] ^b^ | 0.22 | 14.43 | 0.015 | 0.021 | 37 |
| Holms (1996) [46] ^a^ | 0.43 | 20.16 | 0.021 | 0.022 | 2 |
| Walsh & Koshland Jr. (1984, 1985) [48, 49] ^a^ | 0.29 | 16.31 | 0.018 | 0.020 | 15 |
| This work ^a^ | 0.20 | 7.88 | 0.025 | 0.025 | - |

The growth rate (μ), acetate uptake rate (Q_ac_), biomass yield (Y_X/S_), theoretical biomass yield predicted and the percentage difference between experimental and predicted data are shown for each case.

^a^ : Batch culture.

^b^ : Continuous culture.

^c^ : Values of optimal biomass yield were obtained from data interpolation in Fig 5a. For this reason, only studies that reported the corresponding flux distribution are shown.
